# Parenting and Children’s Internalizing Symptoms: How Important are Parents?

**DOI:** 10.1007/s10826-015-0174-y

**Published:** 2015-05-09

**Authors:** Cathy M. van der Sluis, Francisca J. A. van Steensel, Susan M. Bögels

**Affiliations:** Research Institute of Child Development and Education, University of Amsterdam, Nieuwe Achtergracht 127, 1018 WS Amsterdam, The Netherlands

**Keywords:** Parenting, Internalizing, Fathers, Child gender, Young children

## Abstract

Parenting behaviors are associated with children’s internalizing symptoms, however, it is not often examined which factors could possibly influence this relationship. The goals of this study were twofold. One goal was to examine whether the association between parenting and children’s internalizing symptoms would increase if parenting behaviors were assessed behaviorally and in a context where the child displayed specific anxious behaviors. Another goal was to examine whether this relationship was influenced by the age and gender of the child, and by possible parenting differences between mothers and fathers. These questions were examined in a sample of 211 children aged 4–12 years; 140 community children and 71 clinically referred anxious children. Parents completed questionnaires regarding children’s internalizing symptoms and parenting behaviors (positive reinforcement, punishment, force, reinforcement of dependency, and modeling/reassurance). In line with expectations, more punishment and less modeling/reassurance by parents were related to more internalizing symptoms in children. Child gender, child age, parent gender and clinical anxiety status were not found to influence the relationship between parenting and children’s internalizing symptoms. Our results suggest that paternal parenting is as important as maternal parenting with respect to children’s internalizing symptoms, and therefore, fathers could be included in child treatment as well.

## Introduction

Children’s internalizing problems have often been related to parenting practices, particularly parental control and rejection (for reviews see Alloy et al. 2006; Bögels and Brechman-Toussaint 2006; Creswell et al. 2011; Rapee 1997, 2012; Rohner and Britner 2002; Sander and McCarty 2005). Both parental control and rejection (and its subdimensions) are hypothesized to influence children’s internalizing symptoms. For instance, parents who frequently show rejection behaviors (e.g. criticism, disapproval) towards their child may cause the child to develop self-perceptions and schema’s of being incompetent and unacceptable, as well as viewing the world as unsafe and negative, possibly resulting in anxiety (e.g. Bögels and Brechman-Toussaint 2006; Creswell et al. 2011) and/or depression (McLeod et al. 2007a). Children of parents who display overcontrolling behaviors (e.g. unnecessarily assisting children in tasks, controlling their behaviors) may develop perceptions of the self as incompetent, as well as being dependent on parents, having less chances to develop a sense of mastery, feel helpless, and experience the world as out of personal control, which can cause or intensify child anxiety and/or depression (Bögels and Brechman-Toussaint 2006; McLeod et al. 2007a, b). However, the effects found for parenting’s relationship with children’s internalizing symptoms have been modest. That is, four meta-analyses—one examining the relationship between parenting and child depression (McLeod et al. 2007a), two examining the relationship between parenting and child anxiety (McLeod et al. 2007b; van der Bruggen et al. 2008), and one examining the relationship between parenting and child anxiety, depression and internalizing symptoms (Yap and Jorm 2015)—reported effect sizes ranging from *r* = .06 to *r* = .42, depending on what aspects of parenting were measured. As mentioned by McLeod et al. (2007b), the magnitude of these effect sizes are not in line with the postulated important role of parents as proposed in many theoretical models and therefore require an explanation.

How parenting is assessed could be one important reason to possibly explain the modest effect sizes found for the relationship between parenting and children’s internalizing symptoms. In general, parental control and rejection, each existing of different subdimensions, are measured as a whole. However, McLeod et al. (2007a, b) demonstrated that it is more relevant to analyze their subdimensions. For instance, McLeod et al. (2007b) found a large effect for the relationship between autonomy granting, a subdimension of parental control, and child anxiety; and McLeod et al. (2007a) reported a medium effect for the relationship between aversiveness, a subdimension of parental rejection, and child depression. Both these effects were larger than the effects they found for the broad constructs of parental control and rejection. Recent literature now indeed seems to focus more on the specific aspects that make up the broad dimensions of parental control and rejection, while at the same time also widening the focus by including other parenting behaviors such as modelling, inconsistent discipline, and monitoring (Yap and Jorm 2015).

In addition to a possible change in focus on what parenting behaviors are assessed, other considerations should also be taken into account. Almost two decades ago, Rapee (1997) already argued that parenting as assessed with questionnaires should be operationalized behaviorally in order to receive genuine answers. He mentions that there are many negative connotations in parenting questionnaires, which have an impact on how questions are answered. Also, Bayer et al. (2006) explain that contradictory results can easily emerge as different parenting practices can be mixed up depending on the theoretical model that is used by researchers to explain the parenting behaviors. For example, over-involved or protective parenting (social learning theory) can be mistaken for warmth and engaged parenting (attachment theory). Taking these findings together, it seems important to measure parenting in a different way. One potential option is to measure behaviorally operationalized parenting behaviors, not just as single stand alone items, but ideally also in relation to specific child behaviors, to reduce as much bias as possible.

The Child Development Questionnaire (CDQ; Zabin and Melamed 1980) was specifically designed to shed more light on the relationship between parenting and child anxiety and is based on learning principles or, more specifically, functional analyses of child behaviors and parenting behaviors. The CDQ consists of vignettes in which the child is anxious to engage in certain situations. Parents are then asked how they would react to the anxious behaviour displayed by the child, that is: how frequently would they reinforce their child’s anxious behaviour, punish the child for it, force the child into the feared situation, or try to engage the child in the situation by modelling or reassuring the child, or by offering rewards. These parenting behaviors were chosen because previous experimental or clinical research showed a relationship with child anxiety. For example, positive reinforcement of brave behaviors leads to more approach behaviors of the child (Zabin and Melamed 1980). Similarly, Cole and Rehm (1986) found that while both mothers of non clinical children and mothers of depressed children set high standards for their child, mothers of depressed children were less rewarding to their children compared to mothers of non-clinical children. In addition, punitive discipline was found to be positively related to children’s internalizing symptoms, probably through its impact on children’s feelings of safety, lack of parental support and decreased autonomy (Laskey and Cartwright-Hatton 2009). As learning principles are often thought to be important for parents in the treatment of child anxiety (e.g. Manassis et al. 2014; van der Sluis et al. 2012) and depression (Asarnow et al. 2002; Lewinsohn et al. 1990), the scarcity of research on these principles is surprising, as results of these studies could also further inform treatment. Thus, the CDQ may be used as an alternative way of assessing parenting behaviors and contains other parenting constructs, based on operant learning principles, than the questionnaires used in previous research.

As well as how the concept of parenting is measured, other factors related to characteristics of the child and the parent, could have an impact on the association between parenting and children’s internalizing symptoms. One factor to consider is the age of the child. van der Bruggen et al. (2008), for example, incorporated 17 studies that included children as young as 8 weeks old up to the age of 11.9 years and found that child age moderated the relationship between observed parental control and child anxiety. That is, the effects were larger for older compared to younger children. However, the other three meta-analyses (McLeod et al. 2007a, b; Yap and Jorm 2015) did not find any moderating effects of child age on the relationship between parenting and children’s internalizing symptoms. This could possibly be explained by the fact that they included children with different age ranges; i.e., while van der Bruggen et al. included studies focusing on children aged 8 weeks till 11.9 years, Yap and Jorm (2015) included 50 studies focusing on children with a mean age of 5–11 years old, McLeod et al. (2007b) included 47 studies with a focus on children aged 2–18.8 years old, and McLeod et al. (2007a) included 45 studies covering children aged 5.1–18.8 years old. In addition, across all four meta-analyses, only 23 studies were included with a focus on children with a mean age between 4 and 7 years old. It may be these younger children specifically, being highly dependent on their parents and rapidly developing at this same time, who are more easily affected by parenting behaviors (Connell and Goodman 2002).

Another factor that may be important to consider when examining the relationship between parenting and children’s internalizing symptoms is child gender. Girls may show a greater vulnerability to both anxiety and depression, partly due to the different parenting behaviors that girls and boys experience (McLean and Anderson 2009; Nolen-Hoeksema 2001; Zahn-Waxler et al. 2000). With respect to depression, Nolen-Hoeksema (2001) argues that gender role confirmation increases depression among adolescent girls, as girls report to experience more restrictions by, and lower expectations from, their parents than boys. McLean and Anderson (2009) similarly mention that gender-specific role socialization influences the expression of anxiety both in boys and girls. That is, males are encouraged to be assertive and brave, as behaviors of anxiety and withdrawal do not correspond with their gender roles. As such boys may learn active coping to deal with anxiety, whereas girls are allowed to experience anxiety and show dependent and avoidant behaviors. In line, van der Bruggen et al. (2008, meta-analysis) reported a larger relationship between observed parental overcontrol and child anxiety for samples with more girls than boys. However, the three other meta-analyses did not report any moderator effects of child gender on the relationship between parenting and child anxiety, child depression or child internalizing symptoms (McLeod et al. 2007a, b; Yap and Jorm 2015).

A final factor that possibly influences the relationship between parenting and children’s internalizing symptoms is gender of the parent. It is postulated that mothers and fathers have different parenting roles. Mothers are assumed to be more caring with and protective of their children. They promote interpersonal relationships, whereas fathers play with and challenge their children and encourage children’s engagement in the outside world (Bögels and Phares 2008; Bögels and Perotti 2011; Möller et al. 2013). Although results are inconclusive, van der Bruggen et al. (2008) reported a stronger effect size between observed parental control and child anxiety for studies with fathers/both parents participating (*d* = .84) compared to studies that included mothers (*d* = .50). However, the predictor of parent gender was not significant in the final regression model were other predictors were simultaneously included. Nevertheless, a recent meta-analysis supports the different effect of maternal and paternal parenting on child anxiety. That is, paternal not maternal challenging behaviors were negatively related to child anxiety. Also, maternal overcontrol was positively related to child anxiety, whereas paternal overcontrol was negatively associated with child anxiety. Although both relationships were not significant, the difference between mothers and fathers was significant (Möller et al. submitted). No such specific theory or evidence regarding parenting differences based on parent gender exists for child depression (e.g. Phares and Compas 1992) to the author’s knowledge. In keeping with this, no differences were found between mothers and fathers in the relationship between parenting and child depression in the meta-analysis by McLeod et al. (2007a). However, it is important to note that fathers are still largely neglected in research on children’s internalizing problems (Yap and Jorm 2015). When fathers are included in studies, most of the times they are not systematically included but only included as a minority of the total sample (Bögels and Phares 2008). Furthermore, the challenging parenting behaviors that are assumed to be specific to fathers are hardly addressed (Möller et al. submitted). Although not completely overlapping with the concept of challenging parenting, the concept of force, as measured in our study, shows some similarities with challenging parenting and may therefore be more specific to fathers than to mothers.

To summarize, parenting is related to children’s internalizing symptoms, but the effect is only modest. Different factors have been mentioned that could possibly have an impact on this relationship: (1) how parenting behaviors are assessed, that is, which parenting behaviors are measured and how; (2) child age, young children who are developing and learning quickly may be the most susceptible to the influence of parenting; (3) child gender, girls may experience more parenting behaviors associated with children’s internalizing symptoms; and (4) parent gender, fathers may be differentially important in the emergence and/or maintenance of children’s internalizing symptoms.

The objective of this study was to investigate the relationship between parenting and children’s internalizing symptoms, and to explore whether this relationship is dependent on child age, child gender (boys vs. girls) and parent gender (mothers vs. fathers). Although child anxiety and depression are both measured separately and concurrently in studies, they show a large overlap in childhood, and measuring child internalizing symptoms as an outcome measure is therefore of relevance (Yap and Jorm 2015). Also previous studies have combined child anxiety and depression scores due to a high correlation between them (e.g. Low and Stocker 2005). To assess the relationship between parenting and children’s internalizing symptoms, a questionnaire was used that measures parental behavioral responses (i.e. positive reinforcement, punishment, force, modelling/reassurance and reinforcement of dependency) to certain anxious behaviors of the child. Based on previous research (Zabin and Melamed 1980), it was hypothesized that parental punishment, force and reinforcement of dependency would be positively related to child internalizing symptoms, whereas parental positive reinforcement and modelling/reassurance would be negatively associated with child internalizing symptoms. Based on the literature reviewed above, it was further expected that the relationship between parenting behaviors and children’s internalizing symptoms would be stronger for girls than for boys, and for younger compared to relatively older children. We also expected that the parenting behaviors of mothers and fathers would be differentially related to child internalizing symptoms.

## Method

### Participants

The total sample comprised of 211 children (110 boys, 52.1 %) with an age range of 4–12 years (mean age 7.49 years, *SD* = 2.51) and consisted of two subsamples: (a) 71 children who were referred to different mental health care centers due to anxiety issues, and (b) 140 children from a community sample. The mean age of the clinically referred children was 8.15 years (*SD* = 2.36), whereas the mean age of the children from the community sample was 7.15 years (*SD* = 2.52), *p* = .006. Both parents participated in this study for the majority of children (*n* = 166, 78.7 %), for 42 children only their mother participated (19.9 %), and for three children only their father participated (1.4 %). Mean age was 39.26 (*SD* = 4.75) for mothers and 41.96 (*SD* = 5.57) for fathers. Highest educational level for three mothers (1.4 %) was primary education. Fourteen mothers (6.7 %) and 11 fathers (6.5 %) completed lower secondary vocational education. Twenty-eight mothers and 16 fathers finished high school. For the mothers, 14 completed high school at a medium level (6.7 %) and 14 at a higher level (6.7 %). For the fathers, 12 completed high school at a medium level (7.1 %) and 4 at a higher level (2.4 %). Another 55 mothers (26.4 %) and 35 fathers (20.7 %) completed medium vocational education; 56 mothers (26.9 %) and 51 fathers (30.2 %) completed higher vocational education; and 51 mothers (24.5 %) and 52 fathers (30.8 %) completed university. Educational level was missing for one mother (0.5 %) and four fathers (2.4 %).

The clinically anxious children met criteria for at least one anxiety disorder based on criteria reported in the DSM-IV-TR (APA 2000) as established by the staff of the mental health care center. In addition, the Anxiety Disorders Interview Schedule—Parent version (ADIS-P; Silverman and Albano 1996) was administered to the parents of all children (clinically referred children and children from the general population). Based on the ADIS-P, 68 clinically anxious children (95.8 %) and 33 community children (23.6 %) met criteria for at least one anxiety disorder. Of these children (n = 101 of 211) the types of anxiety disorders presented were as follows: specific phobia (*n* = 80, 37.9 %), social anxiety disorder (*n* = 41, 19.4 %), generalized anxiety disorder (*n* = 33, 15.6 %), separation anxiety disorder (*n* = 30, 14.2 %), obsessive–compulsive disorder (*n* = 7, 3.3 %), agoraphobia (*n* = 5, 2.4 %), panic disorder (*n* = 2, 0.9 %), and posttraumatic stress disorder (*n* = 1, 0.5 %). None of the children met criteria for a mood disorder; eight children (3.8 %) met criteria for a form of attention-deficit/hyperactivity disorder and six children (2.8 %) met criteria for oppositional defiant disorder. It should be noted that a selection of this sample was also used in another study (Van der Sluis et al. 2015).

### Procedure

The referred children were part of studies that examined cognitive behavioral therapy for young anxious children ages 4-7 years old (van der Sluis et al. 2012; Van der Sluis et al., submitted) and children ages 8–18 years old (Van Steensel and Bögels 2015). Inclusion criteria of those studies were: (1) children met criteria for at least one anxiety disorder according to the DSM-IV-TR (APA 2000), and (2) at least one parent (if possible mothers as well as fathers) willing to participate in research. Exclusion criteria were: (1) an estimated child IQ below 70 for children aged 8–12 years and below 80 for children aged 4–7 years, and (2) non-treated psychotic disorder, suicidal risk, current sexual or physical abuse. The children from the community sample were recruited by students via their networks, schools, local contacts and sport clubs. Participants, from both the clinical and the community sample, were selected for this current study if at least one parent had completed the Child Development Questionnaire or the Child Behavior Checklist (see instruments) in addition to the ADIS-P interview. Parents and children were informed about the study and signed informed consent (for children only the 12-year-olds). The ethical committee of the research institute Child Development and Education of the University of Amsterdam approved the studies. Assessments took place either at the mental health care centers or at the families’ homes.

### Measures

#### CBCL

The Child Behavior Checklist is a well-known instrument to assess children’s internalizing (and externalizing) behaviors. Due to the different ages of the children, as well as the different places and starting time of data collection, different versions of the CBCL (CBCL; Achenbach 1991; Achenbach and Rescorla 2000, 2001) were used. The internalizing scales show good psychometrics qualities (Achenbach 1991; Achenbach and Rescorla 2000, 2001) and the internalizing scales raw scores of the older (1991) and newer version (2001) of the CBCL show a high correlation (*r* = .98; Achenbach and Rescorla 2001). Raw scores were transformed into T-scores, to enable comparisons between the outcomes of the three different versions of the CBCL.

#### CDQ

Parents completed the Child Development Questionairre (CDQ; Zabin and Melamed 1980). The CDQ consists of vignettes in which a child is showing anxious behavior and parents are asked how they would respond to the behavior of the child. They indicate how often they would use certain behaviors representing punishment, positive reinforcement, reinforcement of dependency, force and modeling/reassurance on a scale from 1 to 5. For example: ‘If my child was afraid of thunder and lightning and wanted to come into bed with me at night, I would most likely: (a) tell him that thunder and lightning were only noises and lights in the far distance and could not harm him while in his own bed [modeling/reassurance]; (b) take him back to his room, put him to sleep, and shut the door [force]; (c) tell him that if he did not sleep in his own bed, he’d be behaving like a baby [punishment]; (d) tell him that if he went back to his own bed, he’d be able to stay up later the next night [positive reinforcement]; (e) let him sleep with me [reinforcement of dependency].

Some modifications have been made to the CDQ since its development in 1980. The questionnaire was updated in 2005 by Perrin. In 2009, Challacombe and Salkovskis developed four additional vignettes related to obsessive compulsive disorder, that were also included in this study, leading to 18 vignettes in total. For the current study, one vignette regarding summer camp was adjusted, as children in our country do not regularly go to summer camps. Internal consistencies of the five subscales were acceptable to good for mothers (positive reinforcement = .86; punishment = .67; force = .83; modeling/reassurance = .83; reinforcement of dependency = .76), and fathers (positive reinforcement = .89; punishment = .76; force = .85; modeling/reassurance = .82; reinforcement of dependency = .76).

### Data Analysis

Since mothers and fathers were nested within the same families, multilevel analyses were used to answer the research questions. This statistic approach has several benefits, that is, it can account for dependencies between respondents and it is able to use all available information (for example, data of mothers can still be included if father data is missing). All continues variables were transformed to standardized normal scores. In this way, the parameter estimates can be interpreted as a measure of effect (Cohen’s *d* for dichotomous variables and *r* for continuous variables). Outliers (i.e. a *Z* value > (−) 3.29) were identified and changed into highest scores not being an outlier. Using Mahalanobis distances, one multivariate outlier was identified. Analyses were run twice (with and without adjusted outliers), however, results did not change and therefore we report on the results with outliers.

To investigate whether parenting behaviors in for children hypothetically anxious situations were related to children’s internalizing problems, and whether this relationship was influenced by parenting differences between mothers and fathers, child gender, and age of the child, while accounting for child condition (clinically anxious children versus children from the general population), level of children’s internalizing problems (CBCL) was used as the dependent variable. Predictors were: parenting behaviors (the five CDQ scales; reinforcement of dependency, force, punishment, modeling/reassurance, and positive reinforcement), parent gender (mothers versus fathers), child age, child gender (boys versus girls), condition (clinically anxious versus control) and interactions between parenting behaviors and these variables. None of the interaction effects were significant in the final model. Therefore, they were dropped from the model that is presented in the results section.

## Results

It was investigated which parenting practices were of importance with regards to children’s internalizing problems and whether this relationship was influenced by other factors (i.e. parent gender, child gender, child age, and condition). Results are displayed in Table 1. Significant main effects were found for the use of punishment and for the use of modeling/reassurance; more parental punishment and less modeling/reassurance in anxiety provoking situations were related to more internalizing problems in children. The size of these parameter estimates (interpretable as *r*) were small; .15 for punishment and −.15 for modeling/reassurance.

**Table 1:** Parameter estimates concerning the effects of parent gender, child gender, child age and parenting practices on child internalizing problems

Parameters	Estimate
Parent gender^a^	.11
Child gender^b^	.13
Child age	−.19***
Condition^c^	1.38***
Positive reinforcement	.02
Punishment	.15**
Force	−.03
Modeling/reassurance	−.15***
Reinforcement of dependency	.04

* *p* < .05; ** *p* < .01; *** *p* < .001

^a^0 = fathers; 1 = mothers

^b^0 = girls; 1 = boys

^c^0 = control children; 1 = clinically anxious children

In addition to the main effects of parenting behaviors, there was a main effect for condition (parameter estimate—interpretable as Cohen’s *d*—of 1.38), indicating that clinically anxious children have more internalizing problems than control children. Also, a main effect for child age occurred, showing that internalizing problems decrease with age (parameter estimate—interpretable as *r*—of −.19). No significant main effect was found for child gender or parent gender, suggesting that boys and girls have similar levels of internalizing problems and that mothers and fathers report similar levels of internalizing problems for their children. No interaction effects were found between parenting behaviors and the other predictors (child age, child gender, parent gender, and condition) and therefore these interactions were dropped from the final model presented here. Such findings, however, suggest that the relationship between parenting behaviors and children’s internalizing symptoms is not influenced by child age, and is not different between boys and girls, fathers and mothers, or clinically anxious children versus control children.

## Discussion

The aim of this study was to examine whether the relationship between parenting and children’s internalizing symptoms would be larger if parenting behaviors were measured in terms of behavior and in a context of children showing anxious behaviors, and when accounting for possible differences between boys and girls, younger and older children and mothers versus fathers. Both clinically anxious (but not depressed) children and community children with and without anxiety disorders were included in the sample. Results were as follows: (I) In line with expectations, it was found that more punishment and less modeling/reassurance were associated with more internalizing symptoms in children; (2) Contrary to expectations, force, positive reinforcement and reinforcement of dependency were not related to children’s internalizing symptoms; and (3) Child gender, child age and parent gender did not have an impact on the relationship between parenting and children’s internalizing symptoms. Each of these findings will be discussed below.

Results showed a positive relationship between punishment and children’s internalizing symptoms, that is, more parental punishment was related to more internalizing symptoms in the child. Punishment as assessed within the CDQ involved behaviors by the parents such as giving negative consequences to the child (e.g. mild spanking), decline of something positive (e.g. not permitted to see friends), belittling the child and making threats. Previous research also reported a positive association between such behaviors and children’s internalizing symptoms, anxiety and depression (e.g. Frye and Garber 2005; Ge et al. 1994; Gershoff et al. 2010; Laskey and Cartwright-Hatton 2009; Low and Stocker 2005; Sheeber et al. 2001). One way in which punitive discipline may affect children’s internalizing symptoms is through its effect on feelings of control as experienced by the child. Chorpita and Barlow (1998) suggest that feelings of personal control over the environment are related to healthy development, especially in case of stress. Parents who punish their children for their anxious behaviors (e.g. mild spanking) are not only irresponsive to their child’s needs, but also control the subsequent consequences for the child. According to Chorpita and Barlow (1998) this combination of parental behavior is detrimental for the child, as it leaves no option for the child to experience a sense of personal control. Eventually this could result in child anxiety via feelings of helplessness and/or child depression via feelings of hopelessness.

Also in line with expectations, the use of more modeling/reassurance was associated with less internalizing symptoms in children. Previous research has shown that parents who model anxious behaviors or who state or expand anxious or depressive cognitions, can contribute to the development or intensification of anxiety and depression in children (e.g. Askew and Field 2008; Creswell et al. 2011; Seligman et al. 1984; Hane and Barrios 2011; Roelofs et al. 2006), but the opposite might also be true. That is, by showing appropriate (brave) behaviors to the child, or by creating smaller steps for children to engage in the feared behavior, or by providing reassuring information to the child, parents may give their children a sense of personal control over their environment as parents provide their children with opportunities to exercise control over their environment. In this way, children can also develop and experiment with new (problem-solving) skills which could further increase their sense of control, resulting in less internalizing symptoms (Chorpita and Barlow 1998).

Contrary to expectations, force (i.e. pushing the child to engage in the feared situation), positive reinforcement (i.e. positive consequences for the child if (s)he engages in the feared behavior) and reinforcement of dependency (i.e. let the child avoid the feared situation) were not related to the internalizing symptoms of children. Force was expected to be associated with more internalizing symptoms, as parents who push their children do not allow them a sense of control over the situation, which could make them feel helpless or hopeless (see Chorpita and Barlow 1998). However, it could also be that force is a too strong or a too negative label for firm behaviors displayed by the parents. That is, by using force, parents ‘push’ their children to engage in situations or behaviors the child sees as frightening, which in reality are not dangerous (e.g. placing the child’s hand on a small harmless puppy). When children are then able to cope with these anxiety provoking situations effectively, this could actually give them a sense of personal control, resulting in less internalizing symptoms. Thus, the relation between force and children’s internalizing symptoms could be curvelinear rather than linear, which could clarify why, on average, there was no significant link to children’s internalizing symptoms.

Also, the results found for positive reinforcement were somewhat surprising, as positive reinforcement as measured with the CDQ links to the concept of contingency management, which is a working mechanism in cognitive behavioral therapy to reduce child anxiety (Manassis et al. 2014). However, using positive reinforcement as an extra motivator to doing exposures in anxiety treatment might work differently from parents dealing with their children’s internalizing symptoms via positive reinforcement. For instance, parental use of positive reinforcement (e.g. giving compliments, presents or candy) might not necessarily influence the internalizing (anxiety) problems of their children as these children will need to learn skills to cope with those situations themselves. Another possible explanation relates to child depression. For depressed children, one explicit step in treatment is to start doing enjoyable activities again (Asarnow et al. 2002; Lewinsohn et al. 1990). As this is already a challenge to them, it is probably even more difficult for them to engage in a situation that they fear, and possible rewards provided by parents may not be enough to engage depressed children in fearful situations.

With respect to reinforcement of dependency (e.g. taking child in bed with parents when there is thunder and lightning), it could be that parents sooth and comfort their child rather than (or next to) increase dependency on their parents. Although increasing children’s dependency on their parents is a realistic consequence as these children do not develop skills to deal with the situation themselves (see Wood 2006), it is also possible that children with internalizing symptoms need this comfort from their parents, as a safe haven from which they can further explore their environment once they are ready. Taken together, the parental behaviors that are included under ‘reinforcement of dependency’ could have both positive and negative consequences. This could explain why, on average, there was no significant association with the internalizing symptoms of the children.

Alongside investigating parenting behaviors beyond parental rejection and control, whether possible mother/father differences would have an impact on the relationship between parenting and children’s internalizing symptoms was also studied. No moderating effect of parent gender was found, suggesting that mothers and fathers are equally important when it concerns the association between parenting and children’s internalizing symptoms. However, it is important to note that certain parenting behaviors, which are postulated to be specific for fathers (i.e. challenging behaviors such as teasing the child, rough and tumble play, physical encounters; Bögels and Phares 2008; Möller et al. 2013) were not assessed in this study. Although the construct of force may overlap somewhat with challenging parenting behavior, we did not find differences between maternal and paternal use of force and its impact on children’s internalizing symptoms. However, challenging parenting incorporates a more playful-based manner of teasing and challenging of the child as a way of encouraging the child to show courageous behavior and extend limits (Bögels and Phares 2008), whereas force means to push the child—unwillingly—in a certain feared situation (Zabin and Melamed 1980). Research has found that paternal challenging behaviors are associated with less child anxiety (Möller et al. submitted) and it may be especially important to include fathers in child anxiety interventions if they show no or only minimal levels of challenging behaviors (Bögels and Phares 2008; Bögels and Perotti 2011). However, more research on paternal parenting behaviors is necessary before firm conclusions on possible mother/father differences and their impact on children’s internalizing symptoms can be drawn. Also of importance is that there is no specific theory with regards to mother/father differences for children’s depressive symptoms to the authors knowledge, although some studies suggest that paternal parenting (e.g. overprotection) might be particular important to adolescent depression (see Sheeber et al. 2001). As internalizing symptoms, rather than anxiety symptoms or depressive symptoms separately, were the outcome measure of this study, this could also have an impact on the results.

Finally, studies showed that girls have higher anxiety and depression rates than boys, and that parents may increase these differences by differential rearing of their sons and daughters (McLean and Anderson 2009; Zahn-Waxler et al. 2000), but this was not supported in our study. This is contrary to van der Bruggen et al. (2008) but in line with McLeod et al. (2007a, b), and Yap and Jorm (2015). Gender differences increase with age for both child anxiety and depression, specifically for child depression in girls and boys of adolescent age (Roza et al. 2003), however, we only took into account children up to and including the age of 12, which may explain why we did not find a difference in the effects of parenting on children’s internalizing symptoms between boys and girls.

In conclusion, despite the efforts that were made, these being: including young children and fathers, examining differences between boys and girls, and using a questionnaire that placed items regarding parenting behaviors in relation to children’s anxious behaviors, the results of our study also indicated only small associations between parenting and children’s internalizing symptoms. These results are in line with other recently conducted meta-analyses (McLeod et al. 2007a, b; Möller et al. submitted; van der Bruggen et al. 2008; Yap and Jorm 2015). This finding does not necessarily mean that parenting is not important for the etiology or maintenance of children’s internalizing symptoms, however—on average—it is not as important as previously thought (McLeod et al. 2007a; Yap and Jorm 2015). Individually, children may differ in their susceptibility to parenting (Belsky 1997). In addition, a child’s (anxious/depressed) temperament may trigger parental rearing behaviors, which may not always be beneficial for the further development of internalizing problems (Bayer et al. 2006). In this way, it might still be important to teach parents how to deal with their child’s internalizing problems once they exist. For instance, Cartwright-Hatton et al. (2005) provided a general parenting skills training to parents of children with externalizing symptoms, but found that the internalizing symptoms of these children decreased as much as the externalizing symptoms. That study results seem to indicate that parents can make the difference in the amelioration of children’s internalizing symptoms, whereas their parenting behaviors may be less important in the emergence or maintenance of children’s internalizing symptoms. It would be interesting to involve fathers more in children’s treatment of internalizing symptoms, as one would then be able to examine possible differences between mothers and fathers in reducing children’s internalizing symptoms.

### Strengths and Limitations

This study had several strengths: (1) The inclusion of young children aged 4–7 years; (2) Many fathers as well as mothers participated in this study; (3) The sample was relatively large and enrolled both clinically referred children as well as children from the general population; (4) A questionnaire was used that assessed different parenting behaviors than the previously measured concepts of parental control and rejection, and this parenting questionnaire also placed parenting behaviors in a context of child anxious behaviors instead of letting parents answer contextless single items. Next to these strengths, this study also had some important limitations: (1) Children’s internalizing symptoms were the outcome measure in this study, but the clinically referred children were all participating in studies examining treatment for anxiety disorders. Hence all clinical children had a primary anxiety disorder, but none of the children had a comorbid mood disorder, although they could have had subclinical levels of depression; (2) The questionnaire measured parenting behaviors in a context of child anxiety, but not child depression; (3) The data used in this study was cross-sectional which means that cause-and-effect relations cannot be established and that children’s internalizing symptoms can also have an impact on maternal and paternal parenting; and (4) Although children with a broad age range were included, no children above 12 years of age were included, as the parenting questionnaire was not appropriate for these older children.
